# Under Pressure: A Microfluidic Chip for Prolonged, Anesthetic-Free Imaging of Neuronal Mitostasis in *Caenorhabditis elegans*

**DOI:** 10.1523/ENEURO.0304-21.2021

**Published:** 2021-09-02

**Authors:** Joy A. Franco

**Affiliations:** 1 Department of Mechanical Engineering, Stanford University, Stanford, California 94305; 2 Department of Molecular and Cellular Physiology, Stanford University, Stanford, California 94305

## Abstract

**Highlighted Research Paper:** Tracking Mitochondrial Density and Positioning along a Growing Neuronal Process in Individual *C. elegans* Neuron Using a Long-Term Growth and Imaging Microfluidic Device by Sudip Mondal, Jyoti Dubey, Anjali Awasthi, Guruprasad Reddy Sure, Amruta Vasudevan, and Sandhya P. Koushika.

To properly form repeated action potentials and achieve synaptic transmission, neurons must maintain electrochemical gradients of sodium, potassium, and calcium ions. This requires both ATP-facilitated pumping of ions across the plasma membrane and buffering of free cytosolic calcium, both of which are key functions of neuronal mitochondria. Neuronal mitostasis—how nerve cells maintain the distribution of mitochondria along their extensive processes—remains a mysterious yet vital element of neural function (Misgeld and Schwarz, 2017). Mitochondria must be produced and trafficked to where they are needed, as well as repaired, degraded, or recycled when damaged. Turnover is a relatively slow process, and so uncovering the regulatory processes that oversee mitostasis requires repeatedly imaging the exact same neuron for prolonged periods (>12 h).

Studies of neuronal mitostasis *in vivo* are technically demanding in mammalian systems where tissues are opaque and anesthetics are generally required to monitor temporal dynamics (Plucińska and Misgeld, 2016). Conversely, the transparent invertebrate *Caenorhabditis elegans* provides an ideal system for noninvasive, anesthetic-free imaging of mitochondria *in situ*. Its highly stereotyped nervous system allows for imaging the exact same neuron through development from hatching to adulthood with minimal morphologic variation among individual animals. *C. elegans* have six touch receptor neurons (TRNs; Fig. 1*A*), that sit just below the thin cuticle or skin of the animal with their long axons extending along the anterior–posterior axis of the worm. At ∼200 nm in diameter, these axons are narrow enough that mitochondria generally move in single file. These features make the TRNs ideal for high-resolution, dynamic, and noninvasive imaging of single neurons. Mondal et al. (2021) shed light on the temporal dynamics of mitostasis using a novel microfluidic chip, inspired by an early platform for immobilizing animals under a pneumatically actuated membrane (Guo et al., 2008), and imaging of single posterior lateral microtubule (PLM) neurons through development (Mondal et al., 2021).

**Figure 1.**
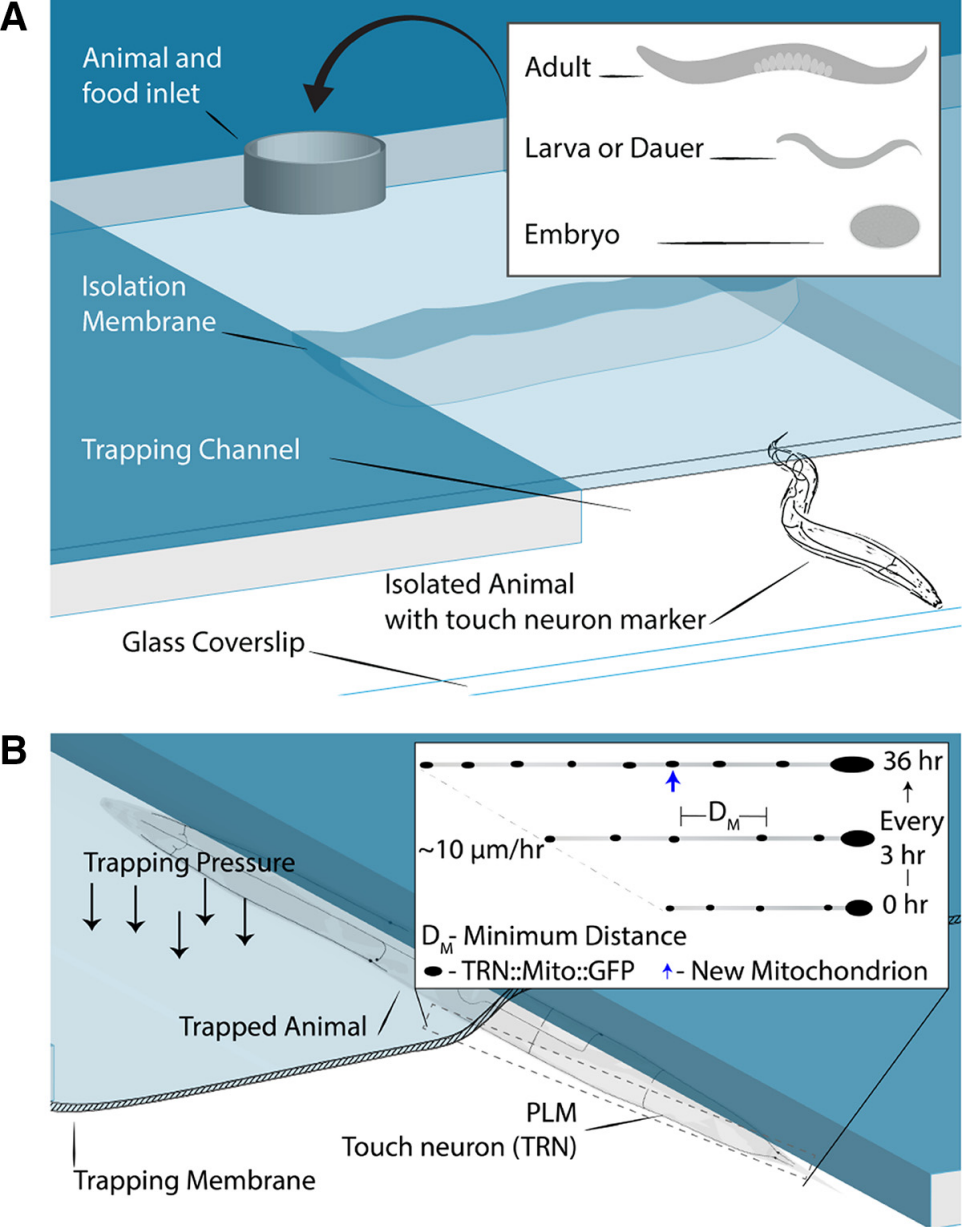
***A***, Simplified cross-sectional view of the microfluidic device showing the inlet that allows the addition of food and animals at any developmental stage. The trapping channel is large enough to allow animals to grow without restriction. The device is bonded to a glass coverslip, allowing for high-resolution imaging. Animals express a TRN-specific, mitochondria-localizing GFP. ***B***, Animals are immobilized for imaging when trapping pressure is applied to the membrane and does not require the use of anesthetics. Any of the TRNs can be imaged because of their morphology and positioning directly below the cuticle of the animal. The authors imaged the PLM once every 3 h through larval development. They found that the PLM elongates at a rate of ∼10 μm/h and that the distance between stationary mitochondria grows with neurite elongation. New mitochondria are added when the distance between stationary pairs exceeds a minimum value of 24 μm.

*C. elegans* develops rapidly compared with other animals, reaching adulthood in 3 d, and the PLM axon elongates at a rate of ∼10 μm/h, as measured by Mondal et al. (2021). Consistent with reports on cell culture and explants (Miller and Sheetz, 2004; Lamoureux et al., 2010; Matsumoto et al., 2020), Mondal et al. (2021) observed that new mitochondria were added to the developing neuron between pairs of stationary mitochondria. These new additions occur at a rate of ∼0.6 mitochondria/h, further highlighting the need for prolonged imaging sessions to observe a sufficient number of events. As expected from earlier studies, the number of mitochondria added through development was proportional to neurite extension, resulting in a uniform distribution that was maintained at nearly all developmental time points. The sole exception was a small, but statistically significant, decrease in mitochondrial density that was observed at the final larval stage. From these experiments, Mondal et al. (2021) ascertained that the distance between adjacent mitochondria needed to reach 24 μm during neurite elongation before a new one is added.

Mitochondria are sensitive to cellular stresses such as those induced by anesthetics, but precisely how anesthesia impacts mitostasis remains unclear (Lewis et al., 2016; Mondal et al., 2021). Mondal et al. (2021) leveraged their microfluidic chip to directly compare mitochondrial turnover in anesthetized *C. Elegans* with that in an immobilized device. Upon imaging the PLM neuron for 20 min in each case, they found that the ratio of moving mitochondria increased approximately twofold in anesthetized animals compared with those immobilized in the device without anesthetics. This differs from previous reports of mitochondrial motility in layer 2/3 pyramidal axons that showed no difference in the percentage of stationary mitochondria between awake and anesthetized mice (Lewis et al., 2016). These discrepancies might be explained by the variations in types of anesthetics used and neurons observed in the respective studies. Mondal et al. (2021) further asked whether anesthesia would affect how motile mitochondria interact with stationary mitochondria that had been photobleached. In all cases, they found that anesthetized animals had more crosses, pauses, and fission events for both anterograde and retrograde moving mitochondria than those that were only device immobilized. Collectively, these findings highlight the utility of studying neuronal mitostasis and mitochondrial motility in experimental systems that allow for anesthetic-free studies.

To demonstrate the utility of the device for other types of subcellular studies, Mondal et al. (2021) measured changes in PLM synapse size through development using the RAB-3 synaptic vesicle marker. As expected, synapse size increased with developmental stage both in animals grown in the device and those grown on traditional agar. However, in the final larval stage there was a statistically significant increase in synapse size in animals that developed in the device compared with animals grown on agar. As the authors point out, this difference in size may be because of the animals being under pressure, since the TRNs are mechanosensitive, but it may also be explained by the fact that animals grown in liquid cultures are longer than agar-grown counterparts. Given that TRNs are insensitive to slowly applied stimuli and static stimuli (Eastwood et al., 2015; Nekimken et al., 2017), additional experiments directly assessing TRN activation under trap conditions are needed to fully resolve this uncertainty. Regardless of either scientific outcome, the device and imaging methods provide a clear entry point for future work studying mitostasis and activity-dependent synapse formation.

Overall, Mondal et al. (2021) present an elegant and simple approach to high-resolution, multiday imaging of the same neuron in single animals that can be adopted by others and extended to studies of phenomena beyond mitostasis. Their results affirm the hypothesis that the uniform distribution of mitochondria is maintained through development via the addition of new mitochondria between docked pairs when a minimum distance is exceeded. Furthermore, this work contributes new information to our limited knowledge of how anesthesia impacts mitochondrial turnover. The current approach is limited in throughput because of the supervision required to identify newly docked mitochondria, but this is a minor barrier that can be overcome as additional baseline data are acquired and automated analysis pipelines are developed. In either case, Mondal et al. (2021) demonstrate that this imaging approach, combined with the convenient size, transparent body, and genetic tractability of the worm, is well suited to directly address key questions of mitostasis such as identifying the signaling events that guide mitochondrial docking. Measuring the temporal dynamics of events like docking as well as mitophagy are critical steps toward understanding the interplay between mitochondrial dysregulation and neurodegenerative diseases (Misgeld and Schwarz, 2017).

## Synthesis

Reviewing Editor: Christophe Bernard, INSERM & Institut de Neurosciences des Systèmes
